# Pleomorphicskin eruptions in a COVID‐19 affected patient: Case report and review of the literature

**DOI:** 10.1002/iid3.382

**Published:** 2021-05-04

**Authors:** Enrico Scala, Luca Fania, Filippo Bernardini, Rodolfo Calarco, Sabrina Chiloiro, Cristiana Di Campli, Sabrina Erculei, Mauro Giani, Marzia Giordano, Annarita Panebianco, Francesca Passarelli, Andrea Trovè, Sofia Verkhovskaia, Giandomenico Russo, Antonio Sgadari, Biagio Didona, Damiano Abeni

**Affiliations:** ^1^ Covid Unit Istituto Dermopatico dellʼImmacolata‐IRCCS, FLMM Rome Italy

**Keywords:** COVID, cutaneous reaction, dermatology, drug reaction, skin, viral exanthem

## Abstract

The coronavirus disease (COVID‐19), during its course, may involve several organs, including the skin with a petechial skin rash, urticaria and erythematous rash, or varicella‐like eruption, representing an additional effect of the severe acute respiratory syndrome coronavirus 2 (SARS‐CoV‐2) infection, as commonly observed in other viral diseases. Considering that symptomatic patients with COVID‐19 generally undergo multidrug treatments, the occurrence of a possible adverse drug reaction presenting with cutaneous manifestations should be contemplated. Pleomorphic skin eruptions occurred in a 59‐year‐old Caucasian woman, affected by a stable form of chronic lymphocytic leukemia, and symptomatic SARS‐CoV‐2 infection, treated with a combination of hydroxychloroquine sulfate, darunavir, ritonavir, sarilumb, omeprazole, ceftriaxone, high‐flow oxygen therapy devices, filgrastim (Zarzio®) as a single injection, and enoxaparin. The patient stopped all treatment but oxygen and enoxaparin were continued and the patient received a high‐dose Desametasone with complete remission of dermatological impairment in 10 days. It is very important to differentially diagnose COVID‐19 disease‐related cutaneous manifestations, where is justified to continue the multidrug antiviral treatment, from those caused by an adverse drug reaction, where it would be necessary to identify the possible culprit drug and to start appropriate antiallergic treatment.

## Graphical abstract

The coronavirus disease (COVID‐19), during its course, may involve several organs, including the skin with a petechial skin rash, urticaria and erythematous rash, or varicella‐like eruption, representing an additional effect of the severe acute respiratory syndrome coronavirus 2 (SARS‐CoV‐2) infection, as commonly observed in other viral diseases. Considering that symptomatic patients with COVID‐19 generally undergo multidrug treatments, the occurrence of a possible adverse drug reaction presenting with cutaneous manifestations should be contemplated. Pleomorphic skin eruptions occurred in a 59‐year‐old Caucasian woman, affected by a stable form of chronic lymphocytic leukemia, and symptomatic SARS‐CoV‐2 infection, treated with a combination of hydroxychloroquine sulfate, darunavir, ritonavir, sarilumb, omeprazole, ceftriaxone, high‐flow oxygen therapy devices, filgrastim (Zarzio®) as a single injection, and enoxaparin. The patient stopped all treatment but oxygen and enoxaparin and received a high‐dose Desametasone with complete remission of dermatological impairment in 10 days. It is very important to differentially diagnose COVID‐19 disease‐related cutaneous manifestations, where is justified to continue the multi‐drug antiviral treatment, from those caused by an adverse drug reaction, where it would be necessary to identify the possible culprit drug and to start appropriate antiallergic treatment.

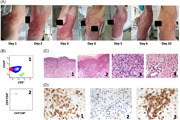

## 1 INTRODUCTION

The coronavirus disease (COVID‐19), initially appeared in Wuhan (China),^1^ is due to an infection by the severe acute respiratory syndrome coronavirus 2 (SARS‐CoV‐2)^2^ often associated with a respiratory failure caused by severe interstitial pneumonia,^3^ and has currently reached a pandemic extent.^4^, ^5^

The disease, during its course, may involve several organs, including the skin with a petechial skin rash,^6^ urticaria and erythematous rash, or varicella‐like eruption, representing an additional effect of the SARS‐CoV‐2 infection, as commonly observed in other viral diseases.^7^

There is currently no specific treatment recommended for COVID‐19 disease. Several medications are being explored such as dexamethasone,^8^ remdesivir,^9^ chloroquine, and hydroxychloroquine^10^, ^11^ (generally in combination with azithromycin), lopinavir‐ritonavir,^12^ Janus kinase inhibitors (baraticinib),^13^ monoclonal antibodies against the interleukin‐6 receptor (tocilizumab and sarilumab),^14^ SARS patient sera,^15^ nonsteroidal anti‐inflammatory drugs,^16^ angiotensin‐converting enzyme 2,^16^ and anticoagulant therapy with heparin^17^ scant, or contrasting data are supporting the efficacy of any of these agents, to date.^18^ Considering that symptomatic patients with COVID‐19 generally undergo multidrug treatments, the occurrence of a possible adverse drug reaction (ADR) presenting with cutaneous manifestations should be contemplated.

## 2 RESULTS

We present the case of a 59‐year‐old Caucasian woman, affected by a stable form of chronic lymphocytic leukemia, admitted to the emergency room due to fever, cough, rhinorrhea, and dyspnea. A marked respiratory failure, bilateral air‐space opacification on lung radiographs, and bilateral, symmetric areas of ground‐glass attenuation on computed tomographic scans, were recorded. A nasopharyngeal swab specimen was collected and tested for SARS‐CoV‐2 RNA by reverse transcription polymerase chain reaction (RT‐PCR), yielding a positive result. The patient was therefore treated with a combination of hydroxychloroquine sulfate (Plaquenil®), darunavir (Prezista®), ritonavir, monoclonal antibodies against the interleukin‐6 receptor (Sarilumb®), omeprazole, ceftriaxone (Rocephin®), high‐flow oxygen therapy devices (Venturi masks), and filgrastim (Zarzio®) as a single injection for neutropenia arising following antiviral therapy. She continued assuming enoxaparin 4000 IU twice a day.

**Figure 1:**
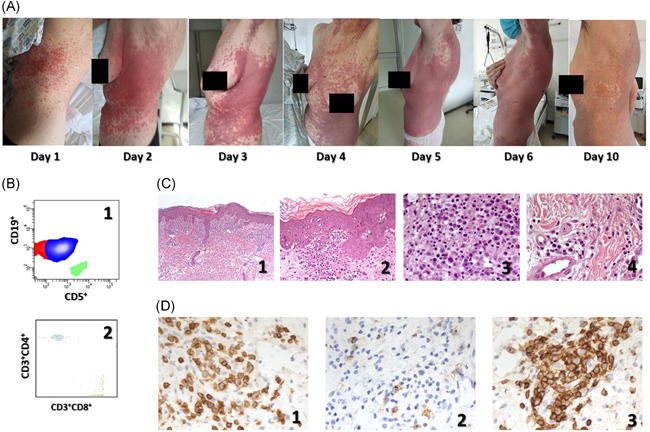
(A) Day by day clinical evolution of skin lesions. Day 1: Widespread and coalescing popular and erythematous lesions with superimposed vesicle or crust are present on the trunk. Day 2: Plaques and papules with erythematous pomphoid appearance are arranged symmetrically on the trunk and limbs. Day 3: Flat and erythematous‐violaceous plaques and papules are located symmetrically on the trunk and limbs. Day 4: Purple‐colored large patches and maculae symmetrically affect the trunk and limbs. Day 5: The skin of the trunk and the root of the limbs is edematous and purplish; the skin of the armpits is spared; the symmetry of the lesion is once again remarkable. Day 6: The skin of the trunk and the root of the limbs is moderately erythematous; the skin of the armpits is spared. Day 10: Skin lesions are healing: postlesional peeling and mild erythema are noted. (B) FACS analysis on PBMC showing the four‐color flow cytometry of CD19/CD5/CD3/CD4/CD8 combination. CD45+ live lymphocytes were gated on forward and side light scatter. (B1) Shows the aberrant overexpression of CD5 by the vast majority of circulating neoplastic CD19+ B cells. (B2) Shows the CD3+CD4+ and CD3+CD8+ distribution in the peripheral blood. (C) Hematoxylin and eosin staining. (C1) Ortho‐ and para‐keratosis, modest edema of the papillary dermis with initial dermo‐epidermal detachment and superficial infiltrate mainly peri‐vascular (original magnification ×5). (C2) Vacuolar alteration of the dermo‐epidermal junction with lymphocyte infiltrate. Presence of some intraepidermal necrotic keratinocytes. In the papillary dermis, there are extravasated red cells and infiltrated lymphocytes, eosinophilic, and neutrophilic granulocytes and some lymphoid blasts (original magnification ×20). (C3) Detail of the infiltrate already described in (C2) showing the presence of red blood cells, lymphocytes, neutrophilic, and eosinophilic granulocytes, blasts (original magnification ×40). (C4) Another detail showing mainly eosinophilic granulocytes infiltrate (original magnification ×40). (D) Immunohistochemistry for CD3 (D1), CD5 (D2), and CD30 (D3) showing that most of the infiltrate in the inflamed skin biopsy is represented by CD3+ and CD5+ T lymphocytes, some of them activated and therefore expressing CD30. Original magnification: ×40

About 20 days later, while respiratory function progressively improved, in the presence of a still positive nasopharyngeal swab, moderately itching widespread and coalescing papular and erythematous lesions with superimposed vesicle or crust, not associated with feverʼs recurrence, appeared on the trunk. In the following days, plaques and papules with erythematous pomphoid appearance emerged symmetrically on the trunk and limbs. Eventually, the same lesions became purple‐colored large patches and maculae symmetrically affecting the trunk and limbs, but sparing the armpits, always with a remarkable symmetry of the lesion (Figure 1A).

A punch biopsy for histological examination was obtained from the patientʼs back on Day 3, and hematoxylin‐eosin stained tissue specimens showed the presence of ortho‐ and para‐keratosis, rare intraepidermal necrotic keratinocytes, edema of the papillary dermis and superficial perivascular, and interstitial infiltrate (Figure 1C), consisting of CD3^+^CD5^+^ T lymphocytes, some of them CD30^+^, having a blastic appearance, very rare CD20^+^ B cells and exceptional CD79a^+^ plasma cells, numerous eosinophilic granulocytes, and scant neutrophilic granulocytes. Such histological findings were suggestive of polymorphic erythema, but the presence of numerous eosinophilic granulocytes was indicative of toxidermic reactions (Figure 1D). Flow cytometric immunophenotyping of peripheral blood lymphocytes confirmed the presence of 94.5% (19,781/µl) CD19^+^ B cells, 87.6% of them beating the T cell marker CD5^+^, aberrantly and commonly expressed in B cell chronic lymphocytic leukemia (Figure 1B). As a consequence, a clear reduction of all the other subsets (CD3^+^ = 3.6%, 412/µl; CD3^+^CD4^+^ = 2.4%, 272/µl; CD3^+^CD8^+^ = 1.1%, 130/µl; and CD3^‐^CD16^+^CD56^+^ = 0.7%, 74/µl) was observed. T cell receptor‐Vβ analysis identified no impairment of the T cell repertoire.^19^, ^20^

The patient received a high‐dose Desametasone (Soldesam®) therapy for 5 days with gradual tapering of dosage for further 2 weeks. The patient resulted negative to the SARS‐CoV‐2 nasopharyngeal swab a week after the rash onset.

Two months later, after obtaining the patientʼs written consent, an allergy study was carried out for β‐lactam reactivity. Skin tests were done by prick, and since negative results were recorded, the intradermal test were performed. The determinants and maximum concentration used were: benzylpenicilloyl polylysine (Allergopen; 5 × 10^−5^ mM/L), a minor determinant mixture containing benzylpenicillin and benzylpenicilloate (Allergopen; 2 × 10^−2^mM/L), penicillin‐G (10.000 UI/ml), and a panel of cephalosporins, including Ceftriaxone (all at 2 mg/ml). The patientʼs serum was tested for the presence of specific IgE to penicilloyl G, penicilloyl V, ampicilloyl, amoxicilloyl, and cefaclor (UniCAP specific IgE; Pharmacia & Upjohn). We also performed patch tests with cephalosporins as previously described.^21^ All tests were negative, and the patient refused a challenge test with ceftriaxone as well as a further investigation with the other possible culprit drugs.

## 3 CONCLUSIONS

Several clinical dermatologic presentations could occur during an ADR, including varicella or morbilliform‐like exanthema, urticaria, erythema multiformis, vasculitis reaction with petechial and purpuric lesions, acral ischemia, and livedo reticularis. Since COVID‐19 could present with all these clinical manifestations, a differential diagnosis between the infectious disease and ADR should be reached. Furthermore, it should be taken into account that many of the symptomatic patients with COVID‐19 are elderly individuals who assume several drugs to control various pre‐existing conditions, thus increasing the risk of ADR.

In Table 1, we differentiate an exanthem triggered by the viral SARS‐CoV‐2 infection from an ADR through the evaluation of clinical, serological, and histological parameters. Marzano et al.^22^ reported that the COVID‐19 exanthem appeared 3 days after systemic symptoms and disappeared after 8 days, without facial or mucosal involvement. In the reported cases of COVID‐19 infection, the itch was mild or absent and cutaneous lesions interested mainly the trunk.^7^, ^22^ Considering laboratory parameters in COVID‐19 disease, elevated levels of lactate dehydrogenase, ferritin, and aminotransferase have been described. Furthermore, high d‐dimer levels and more severe lymphopenia have been associated with higher mortality,^23^ while, on the other hand, atopic status was associated with less severe clinical outcomes.^24^ In case of doubts regarding the cause of the rash, a biopsy would be necessary to confirm the diagnosis. Histological examination of the viral exanthema shows a slightly atrophic epidermis with basket‐weave hyperkeratosis and vacuolar degeneration of the basal layer with enlarged and multinucleate keratinocytes, without lymphomonocytic infiltrate. Otherwise, ADRs present histologically with an interface dermatitis characterized by spongiosis and superficial, or superficial and deep, perivascular and interstitial infiltrate of lymphocytes and eosinophils, sometimes with scanty neutrophils; vacuolar changes at the dermo–epidermal junction with necrotic keratinocytes can often be observed.^25^ However, the histological examination can also be difficult to interpret, as the appearance of viral lesions and ADR may be quite similar. Besides, it must be remembered that in some cases ADRs occur in conjunction with a viral infection, as it happens for example in the morbilliform exanthema due to taking ampicillin during an EBV infection, or in the DRESS syndrome where there is a reactivation of HHV‐6.

**Table 1:** Differential diagnosis between viral exanthem of COVID‐19 and adverse drug reaction

	Viral exanthem (Sars‐CoV‐2 infection)	Exanthem in adverse drug reactions
Onset of cutaneous manifestation^a^	<10 days	1‐>10 days
Respiratory, gastro‐intestinal or other symptoms^b^	+	−
Multidrug therapy	−	+
Symmetric distribution of cutaneous lesions	−	+
Facial or mucosal involvement	−	+
Itch^c^	−	+
Eosinophilia	−	+
Lymphopenia	+	−
Increased total IgE	−	+/−
Increased LDH, ferritin and d‐dimer	+	−
Histology of cutaneous lesions^d^	Viral reaction	Drug reaction

Abbreviations: COVID‐19, coronavirus disease 2019; LDH, lactate dehydrogenase; SARS‐CoV‐2, severe acute respiratory syndrome coronavirus 2.

^a^ Compared with other clinical manifestation or main symptoms of COVID‐19 infection.

^b^ Fever, cough, rhinorrhea, dyspnea, nausea and diarrhea, headaches, myalgia, weakness, coryza, hyposmia, hypogeusia, and pharyngodynia.

^c^ In COVID‐19 infection has been reported no mild itch.

^d^ See description in the text.

We acknowledge that our findings may not be completely novel, but this “N of 1” case report underlines that It is very important to correctly identify the two different etiological situations since they require diverging treatment approaches. In fact, in case of COVID‐19 disease, it would be justified to continue the multidrug antiviral treatment, while in case of ADR it would be necessary to identify the possible culprit drug, to stop as soon as possible the administration of that drug, and to start appropriate treatment (glucocorticoid and/or antihistamine drug).

## CONFLICT OF INTERESTS

The authors declare that there are no conflict of interests.

## AUTHOR CONTRIBUTIONS

Sabrina Erculei and Luca Fania conceived the study, and wrote the manuscript. Filippo Bernardini, Rodolfo Calarco, Sabrina Chiloiro, Cristiana Di Campli, Sabrina Erculei, Mauro Giani, Annarita Panebianco, Andrea Trovè, Sofia Verkhovskaia, Giandomenico Russo, and Antonio Sgadari cared for COVID‐19 patient and provided the clinical data. Francesca Passarelli and Biagio Didona performed histological evaluation. Damiano Abeni performed the statistical analysis and wrote the manuscript.

## Data Availability

The data that support the findings of this study are available from the corresponding author upon reasonable request.
